# Development and evaluation of an indirect ELISA for detection of *Teladorsagia circumcincta* infection in sheep

**DOI:** 10.1186/s12917-021-03042-1

**Published:** 2021-10-12

**Authors:** Jalal Aliabadi, Ehsan Rakhshandehroo, Azadeh Yektaseresht

**Affiliations:** Department of Pathobiology, School of Veterinary Medicine, Shiraz University, P.O. Box 71441-69155, Shiraz, Iran

**Keywords:** *Teladorsagia circumcincta*, Western blotting, ELISA, Sheep

## Abstract

**Background:**

The gastrointestinal helminth, *Teladorsagia circumcincta*, is one of the major health risks and production-limiting diseases in small ruminant populations, particularly in temperate regions. With the increasing importance of disease management and recruited anthelmintic resistant types, accurate approaches are needed for the diagnosis of the infection in the host. Due to uncertain results using faecal examinations, the ELISA method was indicated for the detection of nematode antigenic materials. Despite some promising results, problems were described in terms of test specificity and cross-reactions. Therefore, this study aimed to evaluate the IgG response to worm somatic and excretory/secretory (ES) products using western blot analysis and an indirect ELISA for the detection of *T. circumcincta* infection in sheep.

**Results:**

Based on the immuno-reactivity analysis, immunogenic fractions with molecular weights (MWs) of approximately 60, 75 and 100 kDa were detected in somatic content and two antigens of about 63 and 75 kDa in ES material. Accordingly, a specific product at 75 kDa had the strongest reaction and appeared as the most common antigenic protein. In ELISA, all the sera from the infected sheep revealed the OD rates above the calculated cut-off value with about two-fold greater average. Negative control samples were also specifically recognized with the mean OD rate of about 1/3 of the estimated cut-off value. The cross-reaction test, using rabbit anti-*T. circumcincta* IgG, did not show reactivity with the ES antigens of other prevalent nematodes including *Haemonchus contortus*, *Protostrongylus rufescens* and *Marshallagia marshalli*. In contrast, a strong positive reaction was observed with the somatic antigens of *M. marshalli*.

**Conclusions:**

The results of this study indicated that the indirect ELISA method using the ES content enables distinguishing the *T. circumcincta* infected sheep with high specificity. Those antigenic ES peptides with 63 and particularly 75 kDa MWs should be further investigated due to the potential for serological diagnostic methods and immunoprotective targets in the host.

**Supplementary Information:**

The online version contains supplementary material available at 10.1186/s12917-021-03042-1.

## Background

*Teladorsagia circumcincta* is one of the most prevalent gastrointestinal nematodes (GIN) that infects sheep and goats worldwide. The infection coincides with inflammation in the abomasal tissue leading to changes in the gastric physiological functions [1] and contributes to reduced weight gain and productive indicators [2].

The detection of GIN has been traditionally depended on tracing the eggs in faecal samples by microscopy. Several egg count procedures with subsequent modifications have been reported to estimate the level of infection. However, microscopic assessments have some disadvantages and are often accompanied by unreliable results [3]. Therefore, alternative diagnostic approaches were developed to identify present GIN infections in the host. Among the evaluated methods, the response of the immune system have been mostly investigated [4]. In sheep infected with *T. circumcincta*, protective immunity was associated with the parasite-specific antibodies against adult worms [5] and the established larvae [6, 7]. The isotypes of antibodies play an important role in GIN resistance in sheep, including IgA, IgG1 and IgE [8].

In the past, the potential of the ELISA technique was assessed to detect the antibody response to *Ostertagia* species in field applications. The IgA reaction was comparatively investigated in larval somatic antigen and a fragment of a recombinant protein, disulfide isomerase, in blood, nasal secretions and saliva of the infected ewes [9]. This method was also developed for the detection of IgG antibodies against copro-antigens in faecal preparations [10]. Additionally, the level of IgG specific to *T. circumcincta* crude antigens was described in the milk and blood of goats [11] and experimentally infected lactating ewes [12]. In cattle, ELISA was hopefully performed for the diagnosis of gut-associated nematode infections using recombinant *Cooperia oncophora* protein [13], crude *Ostertagia ostertagi* whole-antigen [14] and *O. ostertagi* copro-antigens containing excretory secretory (ES) products of the worm [15]. Along with the successful or relatively promising results, some studies reported the strong cross-reactions with other nematode antigens among the trichostrongyloid members [16] and the difficulty to obtain crude [13] or somatic antigens [9] with highly standardized preparations. More importantly, improvements may also need to enhance test sensitivity for parasite detection [10].

Due to variations or lack of data on test specificity, more investigations are needed to enhance diagnosis based on tracing the antigens of *T. circumcincta* in the host. Therefore, the objective of this study was first to detect the somatic and ES antigens of *T. circumcincta* in sheep. In addition, those antigens were used to develop a specific ELISA method with high sensitivity or specificity rates. In parallel, the possibility of cross-reactions was evaluated with some prevalent nematodes that are usually found in abomasal and lung tissues.

## Results

### Species confirmation

In this study, the data from molecular evaluations corroborated the morphologic diagnosis. The BLAST search indicated great similarities between the sequence data obtained (MN888739) and available reports for *T. circumcincta* (the phylogenetic relations shown in Additional file 1).

### SDS-PAGE and western blot analysis

**Fig. 1:**
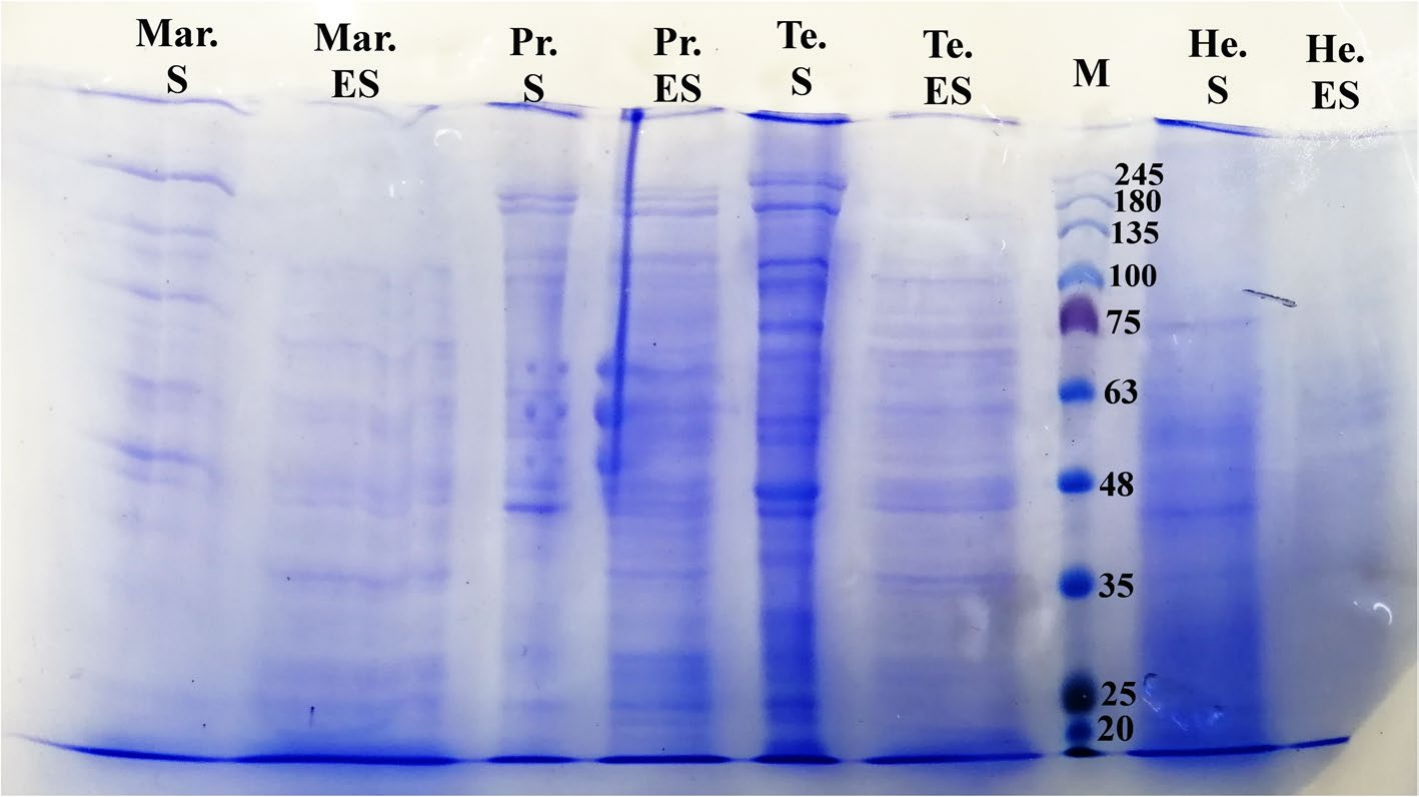
SDS-PAGE analysis of somatic and ES antigens for adult stages of *T. circumcincta*, *H. contortus, P. rufescens* and *M. marshalli*; M: Protein molecular weight marker (Cinnagen®, Cat No. PR901641 [SL7001])

The total protein concentration of somatic and ES antigens were 22.1 and 15 mg/ml, respectively. The SDS-PAGE analysis for somatic antigens revealed 15 protein fractions ranging in size from 20 to 245 kDa, with molecular weights (MWs) of 20, 25, 30, 38, 42 (weak band), 45, 47, 60, 63, 65, 75, 80, 100, 180 and 245 kDa (Fig. 1). In the pattern of ES antigens, the proteins with MWs of 20, 25 (weak band), 28, 35, 48, 50, 63, 68, 75, 80, 100, 135 and 180 kDa could be detected (Fig. 1).

**Fig. 2:**
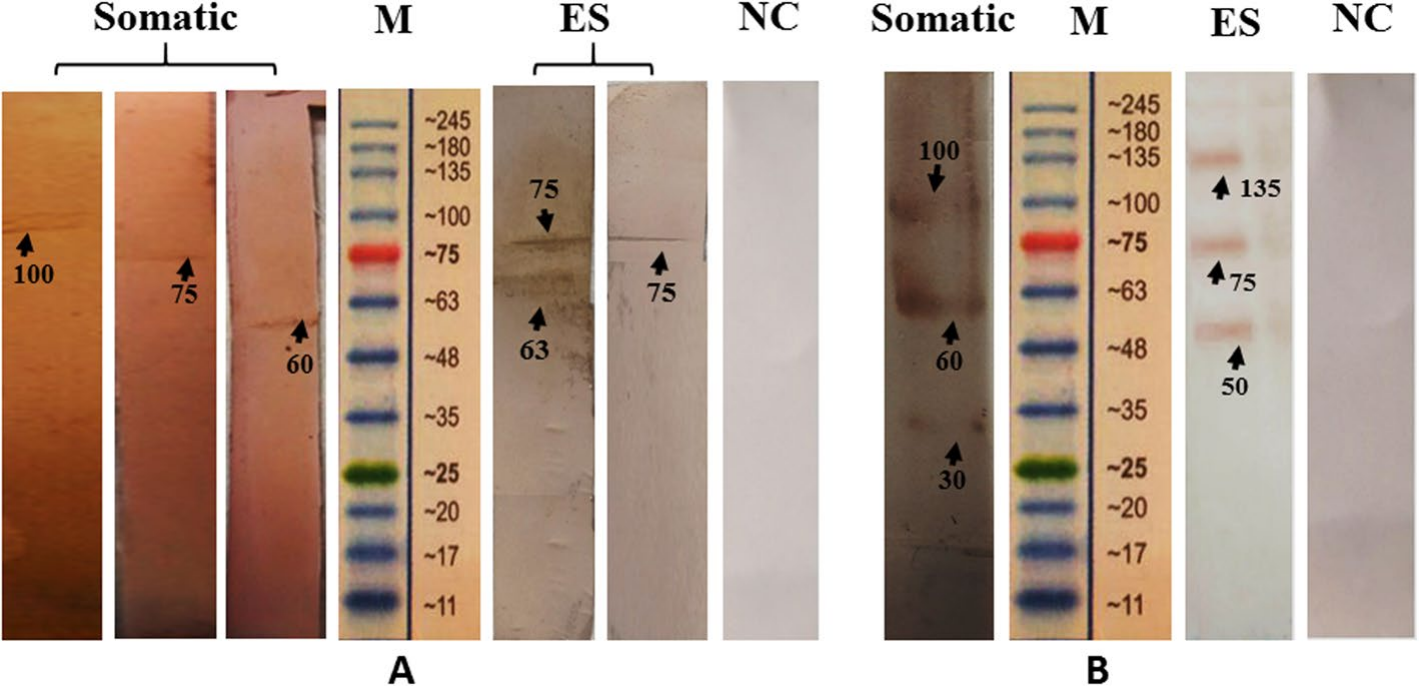
Comparative western blot analysis of immunodominant proteins in somatic and ES content reactive to sera from the *T. circumcincta* infected sheep (**A**) and from hyper immune sera raised in rabbits (**B**). NC: non- immunized rabbit as negative control; M: Protein molecular weight marker (Cinnagen®, Cat No. PR901641 [SL7001])

**Fig. 3:**
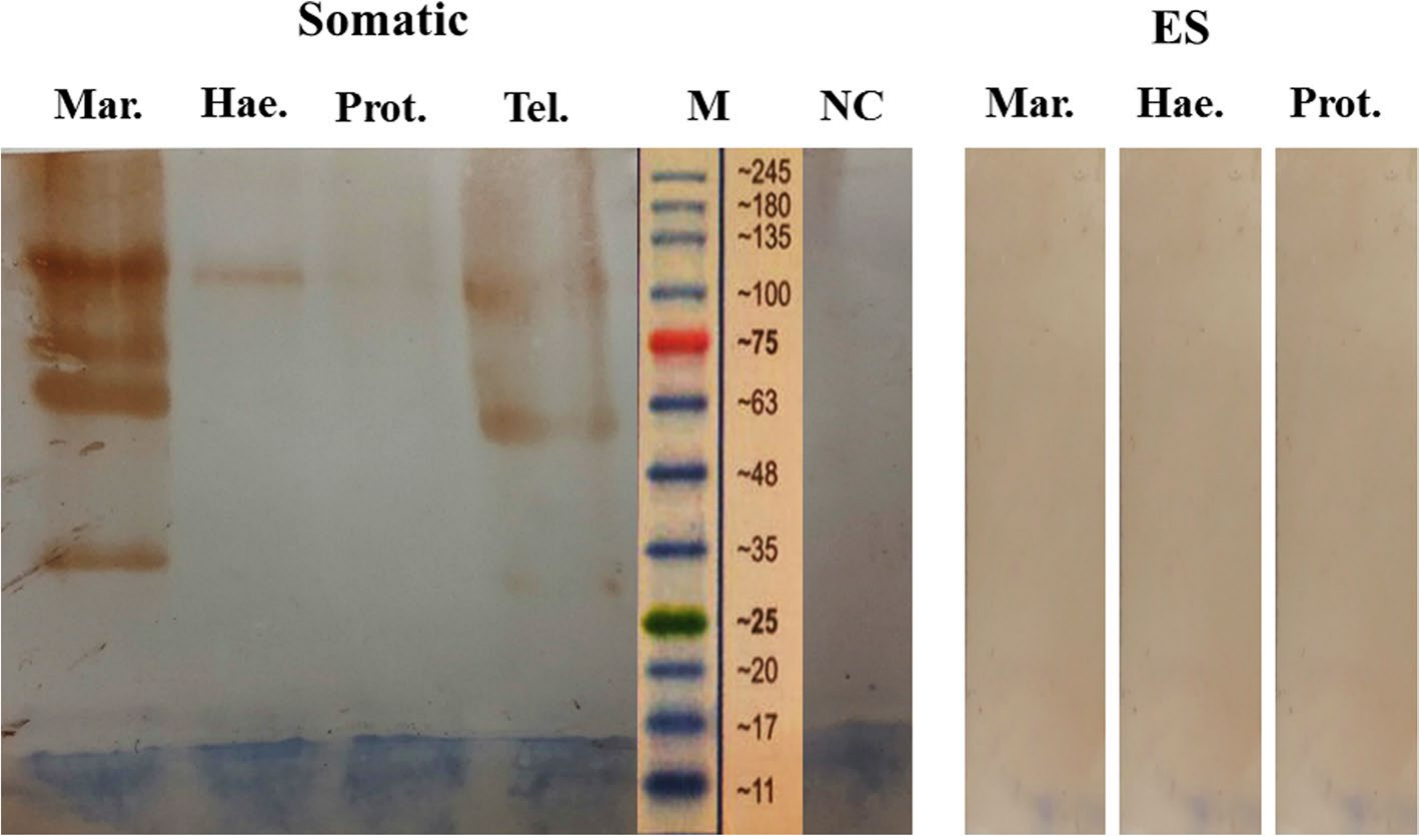
Western blot analysis. Cross reactivity of rabbit hyper immune sera against somatic and ES antigens of *P. rufescens*, *H. contortus* and *M. marshalli.* NC: non- immunized rabbit as negative control; M: Protein molecular weight marker (Cinnagen®, Cat No. PR901641 [SL7001])

The immuno-reactivity of the somatic antigens with the sera from the infected sheep revealed three proteins of about 60, 75 and 100 kDa (Fig. 2A). However, with the hyper immune serum of rabbits, the result was fractions with the MWs of about 30, 60 and 100 kDa (Fig. 3). The immunoblotting analysis with the ES antigens and positive sera of sheep demonstrated only two bands of 63 and 75 kDa (Fig. 2A). One specific band at 75 kDa had the strongest reaction (Fig. 2A). In comparison, fractions with 50, 75 and 135 kDa were obtained using the sera of the immunized rabbits (Fig. 2B). Based on the present electrophoretic patterns, the immune response to somatic antigens of 60 and 100 kDa and ES antigen with 75 kDa were commonly found in samples from sheep and rabbits. In addition, among the somatic and ES protein profiles, the product with 75 kDa MW was the most common antigenic protein. The immunoblotting analysis gave negative reactions with the sera from non-infected sheep.

In western blot analysis, the specificity of rabbit sera against *T. circumcincta* ES antigens was confirmed by a lack of any cross reactions with *H. contortus*, *P. rufescens* and *M. marshalli* ES materials. In contrast, positive anti-somatic sera revealed strong reactivity with the somatic antigens of *M. marshalli* at 35, 63, 75 and 100 kDa. A slight reaction was also found against the somatic antigens of *H. contortus* (Fig. 3).

### ELISA

**Fig. 4:**
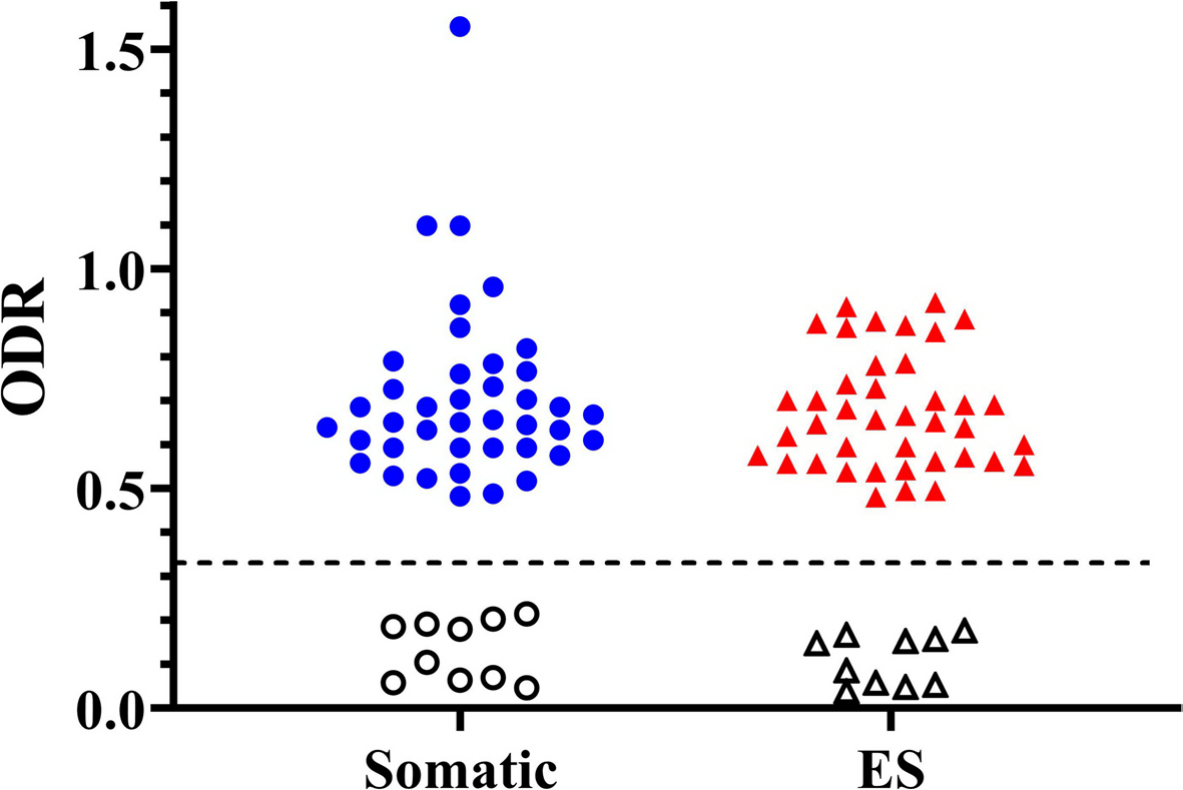
Optical density ratio (ODR) values obtained from sera samples of positive (*T. circumcincta* infected) and negative (non-infected) controls (NC) against somatic and ES products. The dashed line shows the approximate value of estimated cut-off value. Samples under the line are representative of negative controls and those above the line relates to positive ones

The ODR values obtained with the serum samples of the worm-free (negative) and positive *T. circumcincta* specific IgG samples against the somatic and ES antigens are plotted in Fig. 4. The mean ± SEM of ODR values were 0.708 ± 0.03 and 0.674 ± 0.02 for positive samples against somatic and ES antigens, respectively. The respective cut-off values were calculated at 0.348 and 0.328. All the samples from the infected sheep indicated ODR values above the cut-off thresholds. In addition, negative samples registered ODRs with an overall mean ± SEM of 0.12 ± 0.014 within the range of 0.038–0.215. This finding means that the present antigens gave 100% sensitivity and 100% specificity for the diagnosis of the infection.

**Fig. 5:**
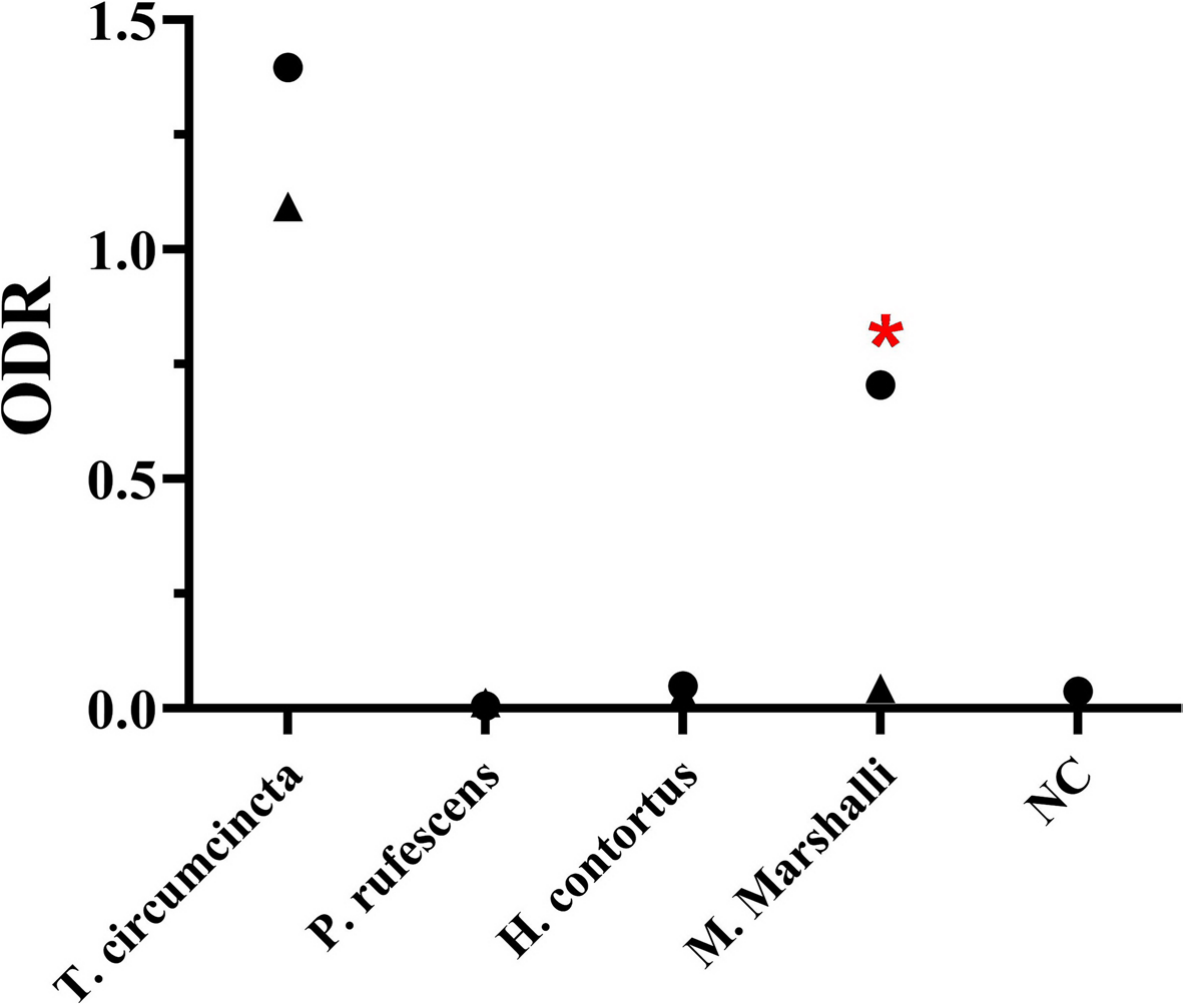
Cross-reactivity by ELISA method. The optical density ratio (ODR) values obtained from rabbit hyperimmune sera against somatic and ES antigens of different prevalent worms. Circles (●) show values against somatic antigens; Up-pointing triangles (▲) represent values against ES material and the red asterisk sign refers to the significant cross-reactivity with the somatic content of *M. marshalli*

According to the observed reactions and the obtained ODR values, no cross-reactions were detected between *T. circumcincta* somatic and ES antigens against the same for *H. contortus*, *P. rufescens* and ES from *M. marshalli*. As previously expected, the relatively high ODR value confirmed a significant reaction with the somatic antigens of *M. marshalli* (Fig. 5).

## Discussion

Based on the results of this study, the ELISA test using the somatic and ES antigens is a sensitive approach for detection of the IgG levels against *T. circumcincta* in sheep. The ES products, compared to somatic antigens, were more specific with no cross reactivity with some other prevalent GIN. Among ES antigens, a peptide with 75 kDa MW was commonly identified in different specimens with negative cross reactions indicating the potential for serological diagnostic methods and immunoprotective studies in the future.

The role of somatic and ES materials has been defined according to the host immune response and protection against GIN infections in ruminants. Numerous secreted proteins, particularly from the ES components, have been characterized in the larval and adult stages of trichostrongylid worms (reviewed in [17]). Studies are more frequently directed on larval secreted proteins with protective potential and applied them for vaccine development [18]. A previous research performed on adult *T. circumcincta* ES products reported peptides varying from 18 to 115 kDa with the most prominent proteins at 20, 29 and 63 kDa MWs [19]. These proteins, also indicated in the results of the present study, are homologs of enzymes involved in energy metabolism reported in *Ostertagia ostertagi* and *C. elegans*. In line with our data, a major protein with 35 kDa MW and a relatively weak protein with 25 kDa MW were described in the ES products of fourth stage larva with Cathepsin F-1 function (Tci-CF-1) [20]. The corresponding CF was also previously explained as the most abundant protein with about 27 to 30 kDa molecule in larval ES from *T. circumcincta* [21]. Despite some similarities, other proteins in the adult stage ES with MWs of about 75 kDa and higher and the whole somatic extract of *T. circumcincta* were not described earlier. Nevertheless, according to the immunoblotting data in this study, ES proteins with MWs of 63 and 75 kDa and somatic antigens with MWs of 60, 75 and 100 kDa could be considered for immunization against and detection of the infection with *T. circumcincta* in sheep.

In this study, the explained indirect ES ELISA was a promising specific method to distinguish between *T. circumcincta* and some other prevalent nematode infections. The cross-reaction among trichostrongylid nematodes has been a limiting factor for test specificity. The levels of reactivity were reported between somatic [11, 16] and ES [10, 22, 23] products derived from *T. circumcincta*, *H. contortus*, *Trichostrongylus colubriformis* and a trematode, *Fasciola hepatica*. Therefore, improvements in the applied method [10], application of purified somatic antigens [24] and use of specific recombinant antigens [17] were suggested to overcome this major problem. However, due to some partial reactivity, the differentiation between *T. circumcincta* and *H. contortus* was remained to be solved. An improved copro-antigen ES ELISA was evaluated for the detection of *T. circumcincta* infection in faecal samples in sheep [10]. A great accuracy was obtained, and the optimized period of exposure to an optimum temperature greatly reduced the cross-reactive signals from all but two cases of *H. contortus*. In another study, the hyperimmune sera against the soluble extract of *T. circumcincta* recognized some protein fractions of *H. contortus* except for a 26 kDa peptide, which was exclusively reactive with homologous antiserum [24]. Such common antigens have been more reported for somatic antigens [23]. In addition to more sensitive results, the ES content acts more specifically to detect *H. contortus* infections [16]. In the present study, a faint reaction was recognized in immunoblotting assay between somatic (but not ES) content of *H. contortus* and *T. circumcincta*. This observation was not confirmed in the ELISA method. In comparison, the significant IgG reactivity was only found against somatic antigens of *M. marshalli*. This result could be expected mainly due to the close phylogenetic relation between *Marshallagia* and *Ostertagia*, both belonging to the Ostertagid nematodes. This is worthy of note that we did not access sera from sheep (experimentally or naturally) mono-infected with prevalent GIN; subsequently the immunized rabbit sera were substituted. Therefore, the assay needs further evaluation using sera samples from the infected sheep from different regions.

The primary aim of the ELISA test was the development of a feasible and specific assay to detect *T. circumcincta* infections. According to the present data, the ODR value seems to be a reliable index for animals with significant infections (with about 1000 or more adult worms). Selection of animals with a threshold of 1000 worm burdens was based on previous studies on *Ostertagia* species. A copro-antigen capture ELISA study using the ES materials produced variable and overlapped OD values in sheep infected with fewer than 1000 *T. circumcincta* [10]. Also, similar unsatisfied results was reported by ELISA on faecal *O. ostertagi* ES content in cattle with natural or experimental infections [15]. On the other side, owing to the increasing concerns to the anthelmintic resistance worldwide, selective (instead of mass) treatment in sheep with heavy infections have suggested for the optimized worm control [25]. In this context, even if it is postulated that ELISA would not discriminate the sera of sheep with low infection intensities and negative controls, the current study contributes to show the practical advantage of this method in line with targeted (selective) treatment to detect those animals harboring much heavier infections. Nevertheless, future studies should be conducted on the outcome of the ELISA method for animals with low burdens and subclinical infections. In addition, the practical value of this method will be increased when the relationship between the abomasal worm counts and the ODR rates could be calculated and statistically analysed.

## Conclusions

This study concluded the functional value of the ELISA test with ES products for screening of infection with *T. circumcincta* in sheep. A strong cross-reaction was observed against somatic antigens of *M. marshalli*, while this reaction was not the case for ES products. According to immunoblot analysis, purified antigenic fractions (like those with MWs of about 63 and 75 kDa) is suggested to be investigated in a broader number of infected and non-infected sheep and goats.

## Methods

### Collection of worm and blood samples

A total of 40 sheep (within 1–2 years of age), with relatively high abomasal infections (harboring about 1000 to 2000 worms), were randomly selected from different flocks referring to the local industrial abattoirs in Shiraz (29.5926° N, 52.5836° E), Fars province, South of Iran. The abomasa were cut and placed in a warm 37 ^°C^ phosphate-buffered saline (PBS). Worms were then recovered, washed three times in PBS, pH 7.2, containing penicillin (10,000 IU/ml) and streptomycin (10 mg/ml) and used for antigen extraction [10]. A number of worms were morphologically identified according to previous descriptions for *T. circumcincta* [26]. Regarding the prevalence of other trichostrongylid nematodes in the sampled area, efforts were made to ensure full recovery of worm burden to exclude abomasa with mixed infection. Prior to the slaughtering process, the peripheral whole blood samples were collected from the jugular vein of animals. Sera were separated from the samples collected from the infected sheep and maintained at − 20^°C^ until use. In addition, blood samples from inspected 10 non-infected animals (under 6 months of age) with no nematode infection were used as negative controls. Sample collections were performed with the consent of the farm owners.

### Molecular diagnosis

Adult *T. circumcincta* worms were randomly selected from at least 2 farms and subjected to genomic extraction using a DNA extraction Kit (MBST, Iran). A primer set was used: F (5′-GCAGACGCTTAGAGTGGTGA-3′) and R (5′-TCCTTGTTAGTTTCTTTTCCTCCG-3′), as described previously to identify the complete rDNA ITS-2 region in ostertagiine nematodes [27]. The PCR reaction mix included 12.5 μl of PCR premix (Ampliqon, Denmark, Cat. No. A180301), 1 μl of each primer, 6.5 μl H_2_O and 4 μl of DNA as template. The cycling program consisted of an initial denaturation at 95^°C^ for 5 min, 94 °C^°C^ for 30 s, followed by 35 cycles of 60^°C^ for 30 s, the extension at 72^°C^ for 30 s and the final extension at 72^°C^ for 10 min. The products were sequenced (ABI 3730 DNA analyzer; Bioneer, Korea) and compared with other available sequences in NCBI using BLAST search. The sequence data was aligned with homologous sequences existing in the GenBank using Clustal W program by M EGA 6 software [28]. The phylogenetic tree was constructed by maximum likelihood (ML) method and analyses was carried out using the Kimura 2-parameter distance estimate [29].

### Preparation of *T. circumcincta* somatic and ES antigens

Somatic antigens were extracted from washed adult specimens. A total of (about) 1000 worms were separately homogenized by tissue grinding, sonicated in 10 mL PBS pH 7.2 and centrifuged at 12000 rpm for 15 min at 4 °C. The extract was filtered through 0.45 mm filters and finally stored at − 20^°C^ until use.

In order to prepare the ES products, freshly isolated adult worms (n ~ 1000) were washed four times in normal saline and subsequently in PBS, pH 7.2, containing penicillin (1000 IU/ml) and streptomycin (1 mg/ml). Worms with high rates of motility were maintained in a sterile culture flask at a density of approximately 100–200 worms/mL of the culture medium (RPMI-1640; Gibco, ThermoFisher Scientific, USA) with penicillin G potassium and streptomycin (1000 IU/ml and 1 mg/ml) and incubated for 24 h in 5.0% CO2 at 37^°C^. Supernatants were collected and centrifuged at 12000 rpm for 15 min at 4^°C^. The supernatant was then dialysed against PBS over a period of 24 h, and concentrated using freeze-drying process (Lyophilisation) (Zirbus, Netherlands). Protein content was estimated by the Bradford method [30] and stored at − 20^°C^ until use.

### Preparation of rabbit polyclonal antisera

**Fig. 6:**
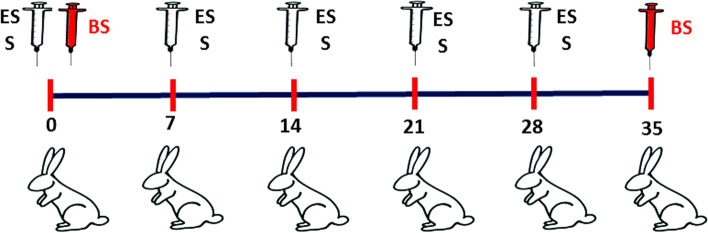
Vaccination protocol for immunization of rabbits against different (somatic(S) and ES) antigenic materials and the respective times for blood sampling (BS)

Ten adult male rabbits of a commercial New Zealand White strain with average weight of 2 ± 0.2 Kg were maintained under constant conditions with access to water and food in the animal house, School Veterinary Medicine, Shiraz University. Rabbits were adapted to new conditions for at least two weeks prior to immunization and divided in to 5 groups. Groups 1 to 4 received somatic and ES antigens of *T. circumcincta*, *H. contortus*, *M. marshalli* and *P. rufescens* and group 5 was defined as control. Blood samples (of about 5 ml) were collected from each rabbit before first injection (day zero) and used as negative controls. According to vaccination protocol (Fig. 6), 300 mg antigen, in a volume of 1 ml of PBS, emulsified with 1 ml of Freund’s complete adjuvant (Sigma, USA) was first injected subcutaneously at day zero. This followed by four boosters comprised of 150 mg antigen in a volume of 1 ml of PBS emulsified with 1 ml of Freund’s incomplete adjuvant (Sigma, USA). Boosters were given at one week intervals. The control group (group 5) was administered with 1 mL of sterile PBS plus 1 mL adjuvant. Rabbits were bled one week after final booster (at day 35), sera were collected and stored at − 20^°C^ until use. The polyclonal hyper-immune sera (containing anti-worm IgGs) were evaluated against somatic and ES antigens by indirect ELISA and Western blotting.

### Electrophoretic analysis

Somatic and ES antigens were separated by sodium-dodecyl-sulfate- polyacrylamide gel electrophoresis (SDS-PAGE) based on Laemmeli’s method [31]. The samples were mixed with an equal volume of a sample buffer and boiled for 5 min. They then were added to each well of a 5% stacking gel and 12% separating gel. Electrophoresis was run at 100 V for 4 h under reducing conditions using electrophoresis apparatus (Paya Pajoohesh Pars, Tehran, Iran). Stained molecular mass standards (Cinnagen®, Iran; Cat No. PR901641 [SL7001]) ranging from 11 to 250 kDa were used. SDS-polyacrylamide gel was stained for protein visualization with 0.05% Coomassie brilliant blue (Sigma, USA.).

### Western blotting

For immunoblotting, proteins were first electrophoresed on 12% SDS-polyacrylamide gel. Western blotting was carried out as described previously [31] with modifications. Proteins were transferred onto a nitrocellulose membrane for 1.5 h. After blocking overnight at room temperature (RT) with 5% skimmed milk in PBS, nitrocellulose membrane stripes were cut, washed with PBST and incubated with serum sample at RT for 1 h. Sera samples from the immunised rabbits and the infected sheep were diluted as 1:50 and 1:10, respectively. After washing, anti-rabbit and anti-sheep conjugate peroxidase (Sigma, USA) diluted in PBS-T (1:2000–1:1000) was added and incubated with shaking for 1 h at RT. Finally, the membrane stripes was washed and placed into a substrate solution 0.05% diaminobenzidine in 50 mM Tris pH 7.4 containing 0.05% H_2_O_2_. (DAB/ H_2_O_2_) (Sigma, USA).

The reactivity of sera from sheep and immunized rabbit against *T. circumcincta* somatic and ES antigens was tested against the same products prepared from prevalent field isolates of *H. contortus*, *M. marshalli* and a lungworm species, *P. rufescens*.

### Enzyme linked Immunosorbent assay (ELISA)

The checkerboard titration for determination of different dilutions of antigen, sera, and conjugate was done [32]. An indirect ELISA (iELISA) was carried out and optimized with serum samples. 96-well microplates were incubated with 100 μl/well of antigen at 1.1 mg/ml for somatic and 7.5 mg/ml for ES proteins in 50 mM carbonate bicarbonate buffer (pH 9.6) at 4^°C^ overnight. After washing three times with PBS containing 0.05% (v/v) Tween 20 (washing buffer), plates were blocked with 200 μl/well of the blocking buffer (PBS at pH 7.2 with 1% Bovine serum albumin) at RT for 2 h. Follow 3 times washings, 100 μl of diluted sheep and rabbit sera 1:2 in 1% BSA were incubated at RT for 1 h. The plates were washed as described above and 100 μl/ well of horseradish peroxidase anti-sheep and anti-rabbit IgG conjugate (Sigma, USA) diluted at 1:5000 were added and incubated for 1 h at RT. The plates were washed three times and 100 μl of the substrate buffer contains (0.02 g Ortho-Phenylenediamine) (Sigma, USA) in citrate buffer and 30% H_2_O_2_ were added to the plate wells. Finally, the optical density (OD) were obtained from an ELISA reader (Immunoskan BDSL, Thermo Lab. Systems, Finland) at 450 nm. All samples were run in duplicate. A pool containing sera of 10 naturally infected sheep with immune-reactivity against *T. circumcincta* (somatic and ES) antigens in western blot test was used as the positive control. Because no serum samples were available from parasite naïve sheep mono-infected with other GINs, the specificity of the method was checked by cross reactivity test using rabbit hyperimmune sera as described earlier.

### Statistical analysis

In order to normalizing the OD estimates in the ELISA, values were quantified as the relative ODR according to the formula: ODR = (OD–N)/(Ps–N), where N and Ps are the mean absorbance values for negative and positive controls. The rates of sensitivity and specificity were evaluated as sensitivity = (true Ps)/(true Ps + false N) × 100 and specificity = (true N)/(true N + false Ps) × 100. In order to determine the best cut-off values, receiver operating characteristics (ROC) analysis was performed and the point showing maximum percents of the sensivity and specificity were considered. The SPSS software (Version 16.0) was used for the statistical analyses and the GraphPad Prism 8 for drawing graphs.

## Supplementary Information

**Additional file 1 **Phylogenetic relations of *T. circumcincta*, recovered from the Iranian sheep, with trichostrongylid species inferred by analysis of the ITS2 rDNA gene using the maximum-likelihood method. The associated numbers represent the percentage of 2000 bootstrap reps and the horizontal distance was proportionated to evolutionary change estimated (scale bar).

## Data Availability

The nucleotide sequence obtained for the ribosomal ITS region of *T. circumcincta* was deposited in the GenBank under the Accession number: MN888739 (http://www.ncbi.nlm.nih.gov/nuccore/1955290121). The datasets generated and analysed during the current study are available in the “Figshare” repository (www.figshare.com).
