# Supplementary figures and images for: Development and evaluation of an indirect ELISA for detection of Teladorsagia circumcincta infection in sheep
Source: BMC Vet Res. 2021 Oct 12;17:326. doi: 10.1186/s12917-021-03042-1 (PMC8510574; doi:10.1186/s12917-021-03042-1)

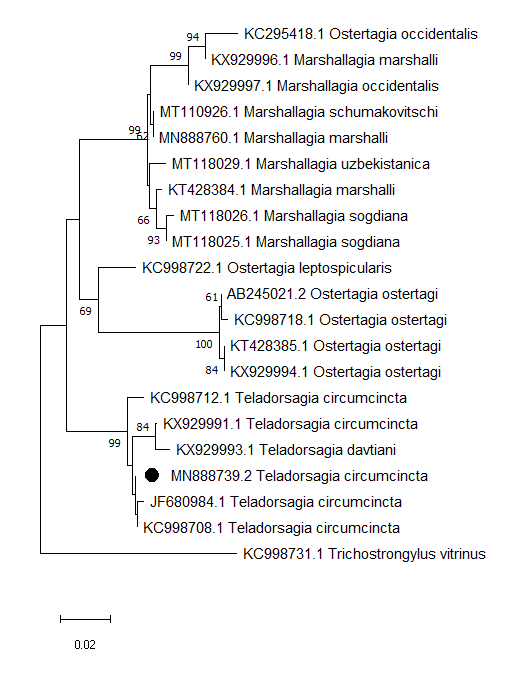

Supplement: Supplementary file 1 — Additional file 1 Phylogenetic relations of T. circumcincta, recovered from the Iranian sheep, with trichostrongylid species inferred by analysis of the ITS2 rDNA gene using the maximum-likelihood method. The associated numbers represent the percentage of 2000 bootstrap reps and the horizontal distance was proportionated to evolutionary change estimated (scale bar). [file 12917_2021_3042_MOESM1_ESM.tif]
